# The influence of atopy and asthma on immune responses in inner‐city adults

**DOI:** 10.1002/iid3.96

**Published:** 2016-02-26

**Authors:** Sujani Kakumanu, Katy Jaffee, Cynthia M. Visness, Amy Dresen, Melissa Burger, Frank R. Witter, George T. O'Connor, William W. Cruikshank, Wayne G. Shreffler, Leonard B. Bacharier, James E. Gern

**Affiliations:** ^1^ School of Medicine and Public HealthUniversity of WisconsinMadisonWisconsin; ^2^ Division of Federal SystemsRho Inc.Chapel HillNorth Carolina; ^3^ Department of Obstetrics and GynecologyJohns Hopkins University School of MedicineBaltimoreMaryland; ^4^ Department of Pulmonary MedicineBoston University School of MedicineBostonMassachusetts; ^5^ Center for Immunology and Inflammatory Diseases and the Food Allergy CenterMassachusetts General Hospital and Harvard Medical SchoolBostonMassachusetts; ^6^ Division of Allergy and Pulmonary Medicine, Department of PediatricsWashington University School of Medicine and St. Louis Children's HospitalSt. LouisMissouri

**Keywords:** Adults, atopy, asthma, cytokines, immune responses, immunology, inner‐city, maternal

## Abstract

Asthma in the inner‐city population is usually atopic in nature, and is associated with significant morbidity and mortality. However, the underlying immune abnormalities that underlie asthma in urban adults have not been well defined. We investigated the influence of atopy and asthma on cytokine responses of inner‐city adult women to define immune abnormalities associated with asthma and atopy. Blood samples were collected from 509 of 606 inner‐city women enrolled in the Urban Environment and Childhood Asthma (URECA) study. We tested for associations between atopy and asthma status and cytokine responses in peripheral blood mononuclear cells incubated ex vivo with a panel of innate and adaptive immune stimulants. Atopic subjects had heightened Th2 cytokine responses (IL‐4, IL‐5, IL‐13) to cockroach and dust mite antigens, tetanus toxoid, and phytohemagglutinin (*P* < 0.05 for all). Differences in cytokine responses were greatest in response to stimulation with cockroach and dust mite. In a multivariate analysis, atopy was broadly related to increased Th2‐like responses to all antigens and PHA, while asthma was only weakly related to mitogen‐induced IL‐4 and IL‐5 responses. There were few asthma or allergy‐related differences in responses to innate stimuli, including IFN‐α and IFN‐γ responses. In this inner‐city adult female population, atopy is associated with enhanced Th2 responses to allergens and other stimuli, and there was little or no additional signal attributable to asthma. In particular, these data indicate that altered systemic interferon and innate immune responses are not associated with allergies and/or asthma in inner‐city women.

## Introduction

Asthma is more prevalent in the inner city and is associated with increased morbidity and mortality as compared to the general population 1, 2, 3. Unique features of the urban environment, such as high exposures to certain allergens such as cockroach and mouse, pollutants and stress likely influence the phenotype and the associated immune profile of urban asthma. Asthma is closely linked to atopy in inner‐city settings; however, the immunologic changes that underlie and potentially differentiate atopy and asthma in this population are incompletely understood.

Altered innate and diminished antiviral immune responses can be a risk factor for atopy and wheezing in early life and quantitative measures of atopy significantly influence asthma onset and phenotype in early childhood 4, 5, 6. There is less consistent information about systemic cytokine responses, allergy, and asthma in young adults with established asthma 7, 8, 9, 10. Young suburban adults with nonallergic or allergic asthma may have enhanced PBMC mitogen‐induced Th1 and Th2 responses, respectively 9. Analysis of cells from the airways of adults with asthma has revealed a bias toward Th2‐driven cytokines, including IL‐3, IL‐4, IL‐5, IL‐9, and IL‐13 11, 12. Similarly, studies of mononuclear cells and cloned T cells from peripheral blood of atopic asthmatic adults and children have described a Th2 bias to antigen 7, 10. In addition, there is evidence that deficient interferon responses in asthma may promote virus‐induced wheezing illnesses, and in children, the development of asthma 8, 13, 14, 15, 16, 17. Further investigation of the immune response to microbial and antigenic stimuli in allergic and asthmatic adults are needed, particularly in the urban population, where asthma prevalence and morbidity is especially high. Furthermore, exposure to allergens especially prominent in the inner city, such as cockroach, may uniquely influence the relationships between atopy, asthma, and the immune responses in the inner‐city population.

The Urban Environment and Childhood Asthma (URECA) is a birth cohort study involving families from four US urban centers (St. Louis, MO, New York, NY, Baltimore, MD, and Boston, MA) recruited between February 2005 and March 2007. As part of this protocol, mothers of children enrolled in the study had blood samples drawn and analyzed for patterns of cytokine secretion. This large data set provided an excellent opportunity to further define the innate and adaptive cytokine responses to allergen and infection in urban women with atopy, asthma, both or neither. We hypothesized that atopy, particularly to indoor allergens, would be associated with increased antigen‐induced Th2‐like cytokine responses, and that these responses would be especially prominent in women with allergic asthma. We also expected that asthma in this population would be associated with deficient Th1‐like cytokine (IFN‐γ and IL‐12p40) and reduced type 1 interferon responses in response to viral and microbial stimuli, as reported in other studies 18, 19, 20.

## Materials and Methods

### Study population

Details of recruitment, enrollment procedure, and timelines for the URECA study have been described previously 21. One thousand eight hundred fifty mothers were screened, 776 met eligibility, and 557 were enrolled (560 infants; three sets of twins were enrolled). Inclusion criteria were as follows: residence in a census tract with at least 20% of residents below the poverty level, at least one parent with a history of allergic rhinitis, eczema and/or asthma, and birth of the enrolled child at ≥34 weeks gestation. Additionally, a control group was recruited from families without allergies or asthma. For this group, 240 families were screened, 70 families met eligibility, and 49 families were enrolled. For the present study, which specifically investigated the immune profile of adult asthmatics, peripheral blood mononuclear cell cytokine responses from mothers participating in the URECA cohort were analyzed. The Human Subjects Committees at the University of Wisconsin and the four clinical sites approved the study.

### Collection of peripheral blood samples, cell stimulation, and cytokine assays

Of the 606 mothers enrolled, 509 had a successful collection of peripheral blood at one of three postpartum visits: 85% were drawn at the 1 year postpartum visit, 7% at 2 years, and 8% at 3 years. Mononuclear cells were separated by density gradient centrifugation within 16 h of collection and cells (1 × 10^6^ cells/1 mL) were incubated for 24 h in the presence of medium alone and the innate immune stimulant panel which included the following: lipopolysaccharide (LPS) 0.1 μg/mL, polyinosinic‐polycytidylic acid (polyIC) 25 μg/mL, peptidoglycan (PG) 1.25 μg/mL, CpG‐A 1 μg/mL, respiratory syncytial virus (RSV) 500 syncytia forming U/mL, and rhinovirus (RV) 2.5 × 10^6^ plaque forming U/mL. The adaptive immune responses were evaluated by incubation with antigens (cockroach extract [CR] 10 μg/mL, dust mite extract 10 μg/mL [*Dermatophagoides pteronyssinus*], or tetanus toxoid [TT] 5 μg/mL) or medium alone for 5 days. Polyclonal responses were assessed following a 24 h incubation with either phytohemagglutinin (PHA) 15 μg/mL or paired monoclonal antibodies specific for CD3 and CD28 (MAB 0.01 μg/mL each). After stimulation, cell supernatant fluids were collected, divided into aliquots, frozen at −80**°**C, and sent to the central processing center in Madison, WI for cytokine analysis. All clinical centers used the same lots of stimulants, and technicians attended periodic training sessions, and standardized specimens were processed yearly to assess quality control as previously described 21, 22. The concentrations of the stimulants were selected to cause approximately half‐maximal cytokine responses, as indicated by preliminary experiments. Cytokine analysis was performed by multiplex ELISA assays (Upstate Biotechnology, Lake Placid, NY; and Beadlyte, Millipore, Inc., Billerica, MA). Sources of stimulants and limits of detection of the cytokine assays have been described previously 21, 22, 23.

The blood samples were also analyzed for total IgE levels as well as specific IgE (fluoroenzyme immunoassay [FEIA], Phadia) to prevalent aeroallergens (birch or oak, ragweed, timothy grass, *Dermatophagoides farinae*, *D. pteronyssinus*, dog epithelium, cat dander/epithelium, German cockroach, mouse urine protein, and *Alternaria alternata*) to screen for sensitization to allergens. Study participants with at least one positive test to an aeroallergen (≥0.35 kU/L) were classified as atopic.

### Allergic and asthma history

Asthma history of the adult female subjects was obtained through surveys that were administered at prenatal visits. Atopy was defined as having one or more positive tests for specific IgE to any allergen. Current asthma was determined by patient report of current diagnosis of asthma, together with either wheezing in the past year or the use of the following asthma medications: albuterol, inhaled corticosteroids, or ICS/LABA combination therapy within the past year.

### Statistical analysis

Linear regression was used to compare immunologic responses between mothers with and without atopy and asthma. These models were adjusted for site, as cytokine responses varied by site, as well as log (10) transformed response to the media control at 48 h. Race/ethnicity of the mother was also examined as a potential confounder; however, we cannot simultaneously control for site and for race/ethnicity in our models due to collinearity (two sites are >95% African American). We controlled for site in our models as site differences were found in previous research 23. As a sensitivity analysis, we controlled for race/ethnicity instead of site, and saw no differences in the results. Additional potential confounders were examined (race, birth season of child, season of blood draw, birth season of mother), but these were not related to cytokine responses. In addition, we controlled for the tobacco use in the prior year (39.8% of subjects) and found no relation between smoking and systemic cytokine responses. Wilcoxon Rank‐Sum tests were also performed as nonparametric conservative comparisons and yielded similar results. Cytokine data was log‐transformed prior to analysis due to the skewed nature of the immune responses. All statistical analyses were performed using SAS 9.3 and R 3.1.2.

## Results

### Study population

Our study included 509 inner‐city women. Data regarding asthma status and medication use was unavailable for one subject. IgE laboratory data were available for 504 subjects. The average age at recruitment during pregnancy was 24 ± 6 years (Table 1). Approximately three‐fourth of the participants were African American (72%), 20% were Hispanic, and the remainder identified themselves as white, mixed, or other. Nearly 40% of participants report a recent history of smoking. Most of the women (69%) reported having an annual family income of <$15,000 dollars. A majority of subjects (68%) were atopic, and over one‐third of participants were sensitive to cockroach and/or dust mite. Thirty‐six percent of subjects reported a current asthma diagnosis. Thirteen percent of subjects were using inhaled corticosteroids, and 9% reported an oral corticosteroid burst in the past year. One hundred twenty‐two participants (24%) had neither atopy nor current asthma (Table 1).

**Table 1:** Participant characteristics

	(*N* = 509)
Age of mother at recruitment	24.4 (5.9)
Mother smoked in the past year	200 (39.8%)
Household income <$15,000	353 (69.5%)
Asthma status	185 (36.4%)
Medication use in past 12 months	
Albuterol	173 (34.1%)
Inhaled steroids	68 (13.4%)
Oral steroids	47 (9.3%)
Advair	60 (11.8%)
Montelukast	24 (4.7%)
Any positive specific IgE	342 (67.9%)
Positive cockroach specific IgE	188 (37.3%)
Positive dust mite specific IgE	170 (33.7%)
Atopy and current asthma	
Atopic asthma	143 (28.4%)
Atopic no asthma	199 (39.5%)
Nonatopic asthma	40 (7.9%)
Nonatopic no asthma	122 (24.2%)

### Peripheral blood cytokine responses

**Figure 1:**
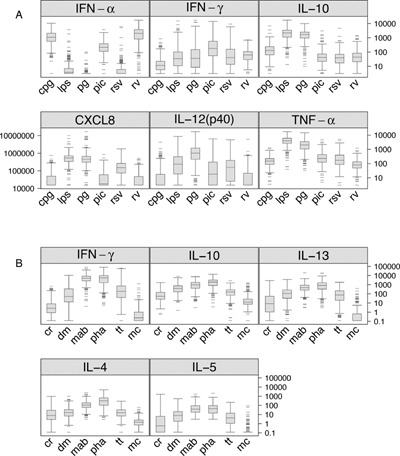
PBMC cytokine responses. PBMC were incubated with stimuli for innate (A) and adaptive and polyclonal responses (B). Cytokine secretion is reported in pg/mL with median values indicated by horizontal bars. CpG, type A CpG; LPS, lipopolysaccharide; PG, peptidoglycan; PIC, poly‐IC; RSV, respiratory syncytial virus; RV, rhinovirus and (Fig. 1B) CR, cockroach; DM, dust mite; MAB, monoclonal Ab (anti CD3/anti CD28); PHA, phytohemagglutinin; TT, tetanus; MC, media control. Cytokine responses to antigens were significantly greater than those of unstimulated control cells (*P* < 0.05 for all).

Peripheral blood cytokine responses to the innate and adaptive stimuli panels were examined. Incubation with innate stimulants induced cytokine responses that were stimulus specific (Fig. 1A). For example, CpG, RV, and PolyIC induced the greatest IFN‐α response, and PG and LPS were particularly potent at stimulating CXCL8, IL‐10, IL‐12p40, and TNF‐α.

Incubation with adaptive stimuli induced significant secretion of IFN‐γ, IL‐10, IL‐4, IL‐5, IL‐13 compared to cells incubated in medium alone (*P* < 0.01, Fig. 1B). Comparing antigen‐specific stimuli, tetanus, and dust mite proteins induced greater cytokine secretion than cockroach, while the polyclonal stimuli PHA and paired CD3+/CD28+ monoclonal antibodies induced the greatest cytokine secretion.

Potential differences in cytokine production (IL‐4, IL‐5, IL‐13, IL‐10, and IFN‐γ) were analyzed by season of blood draw, parity, smoking, and age. No consistent significant effects were observed for these variables.

### Effects of atopy on specific cytokine responses

**Figure 2:**
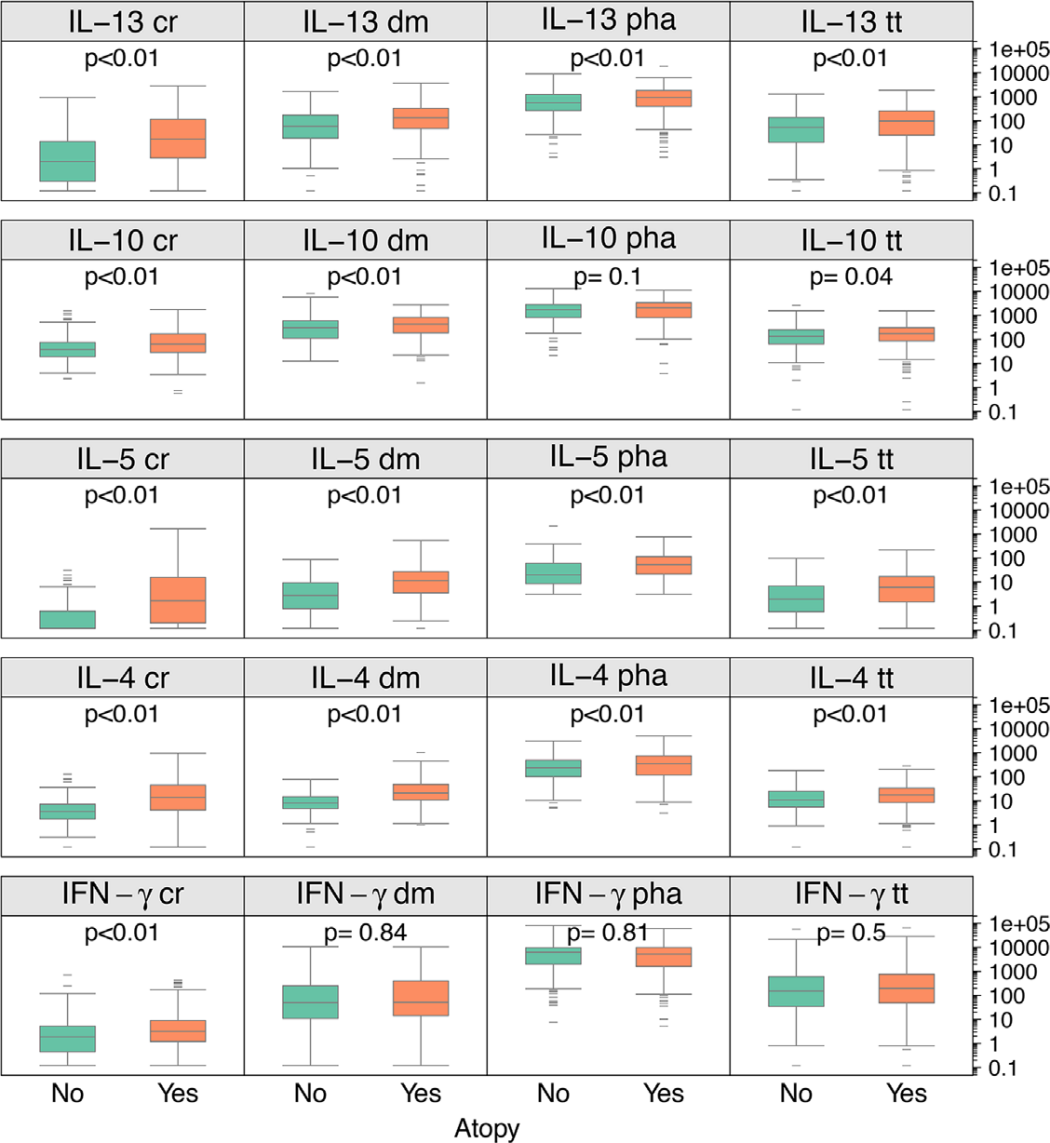
Effects of atopy on cytokine responses. IL‐4, IL‐10, IL‐5, IL‐13, and IFN‐γ, responses (pg/mL) to selected antigens and mitogens are illustrated for nonatopic (green) versus atopic (orange) subjects.

Atopy was associated with consistently increased Type 2 cytokine responses to both antigen‐specific and polyclonal stimuli (Fig. 2). For example, subjects with atopy had significantly greater IL‐13 responses to PHA (median values 934 vs. 560 pg/mL, respectively) and to antigen specific stimuli (median values: cockroach, 17 vs. 2; dust mite, 135 vs. 59; tetanus 98 vs. 53 pg/mL) as compared to nonatopic subjects. Atopy was also associated with greater IL‐10 responses to antigens (median values: cockroach, 64 vs. 38; dust mite, 432 vs. 304; tetanus 172 vs. 133 pg/mL) but not PHA. Type 1 responses were less affected by atopy, as only CR‐induced IFNγ secretion was significantly increased compared to the nonatopic group.

**Figure 3:**
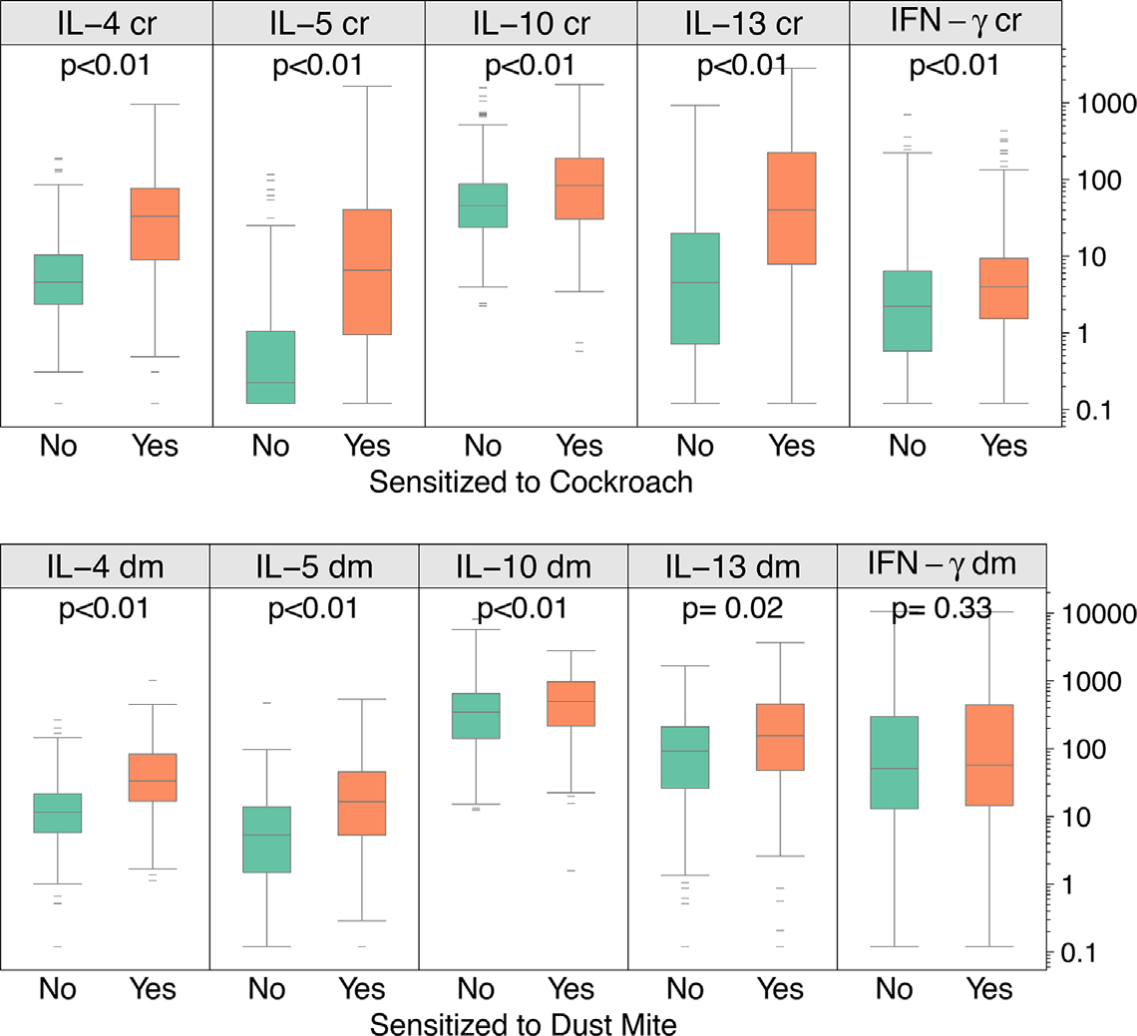
Antigen‐specific cytokine responses in subjects sensitized to cockroach and dust mite. PBMC responses to cockroach extract are grouped according to the presence (orange) or absence (green) of cockroach‐specific IgE. Likewise, PBMC responses to dust mite extract are grouped according to the presence (orange) or absence (green) of dust mite‐specific IgE.

Allergen‐specific cytokine responses were particularly increased by sensitization to the same allergen. In women who were sensitized to cockroach, Type 2 cytokine responses (IL‐4, IL‐5, IL‐10, and IL‐13) to cockroach stimulant were increased (Fig. 3). Subjects sensitized to dust mite showed similar increases for these cytokines in response to dust mite but no differences in IFN‐γ secretion (*P* = 0.33, Fig. 3). Interestingly, in contrast to dust mite sensitive subjects, cockroach sensitive subjects had significant increases in cockroach induced IFN‐γ responses (Fig. 3) indicating a combination of heightened Types 1 and 2 cytokine responses to cockroach in sensitized individuals.

### Testing for separate and combined effects of atopy and asthma

**Figure 4:**
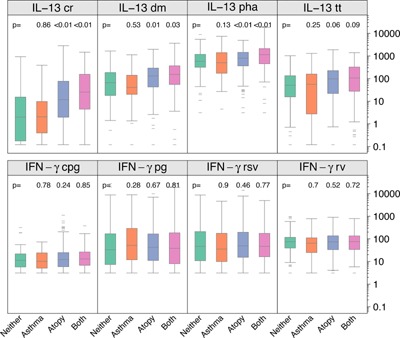
IL‐13 responses to antigen and mitogen stimulation and IFN gamma responses to viral and TLR‐ligand stimulation according to atopy and asthma status. *P* values reported are from a linear regression model and show differences between the following groups: “Asthma” (asthma but no atopy, orange), “Atopy” (atopy but no asthma, blue) and “Both” (asthma and atopy, pink) against the reference group “Neither” (no asthma and no atopy, green).

We next examined the separate and combined effects of allergy and asthma status on innate and adaptive immune responses. To determine whether the heightened Type 2 cytokine responses were more closely related to atopy versus asthma status, we compared subjects with neither atopy nor asthma (“Neither”) to each of the following groups: asthma without atopy (“Asthma”), atopy but no asthma (“Atopy”), and atopy with asthma (“Both”, Fig. 4). Compared to the Neither group, allergen‐induced Th2 responses were increased in the Atopy and Both groups, but not the Asthma group. For example, the median CR‐induced IL‐13 responses were higher in the Atopy (12 pg/mL) and Both (25 pg/mL) populations compared to the Asthma (2 pg/mL) and Neither (2 pg/mL) groups. Furthermore, group‐specific differences in IL‐13 responses were greatest in response to allergens (DM and CR), and there were smaller but still statistically significant increases in response to PHA, and borderline significant trends for responses to TT (Fig. 4). Findings for IL‐4 and IL‐5 responses were similar to IL‐13 responses (data not shown).

**Figure 5:**
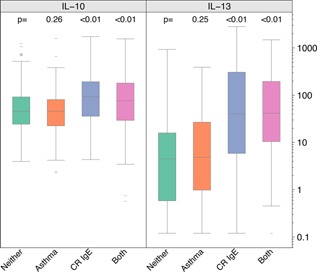
Cockroach‐induced cytokine responses according to the presence of asthma alone (orange), positive CR‐specific IgE alone (“CR IgE”, blue), both asthma and CR‐specific IgE (pink), and neither asthma or atopy (green). Data shown for IL‐10 and IL‐13 only; similar response patterns were observed for IL‐4, IL‐5, and IFN‐γ.

Next, we limited the definition of atopy to those sensitized to CR, and tested whether CR‐specific cytokine responses were similarly related to atopy and asthma. Subjects with cockroach‐specific IgE had greater CR‐stimulated cytokine responses as compared to subjects with neither cockroach sensitization nor asthma (Fig. 5). CR‐specific IL‐10 and IL‐13 responses were increased in CR‐sensitive subjects, with or without asthma, whereas asthma alone did not result in increased responses compared to participants without asthma or CR sensitivity.

When atopy and asthma were analyzed as separate predictors in a multivariate model, atopy showed consistent and statistically significant associations with cytokine expression in response to most adaptive stimulants (Table 2) and some innate stimulants (Appendix Table A1). In contrast, asthma status showed significant relationships only to PHA‐induced IL‐4 and IL‐5, RSV‐induced IL‐10, and RV‐induced CXCL8, and the strength of these associations was comparatively modest (Table 2 and Appendix Table A1). Furthermore, there were no significant interactions between atopy and asthma in this population.

**Table 2:** Results from multivariate model relating asthma and atopy to cytokine responses to adaptive and polyclonal stimulants.*

Cytokine + stimulant	Asthma N = 183/503 (36%)	Atopy N = 342/503 (68%)
IL‐13 CR	1.42 (*P* = 0.15)	**3.95 (*P* < 0.0001)**
IL‐13 DM	0.93 (*P* = 0.68)	**1.86 (*P* = 0.0006)**
IL‐13 PHA	1.22 (*P* = 0.11)	**1.47 (*P* = 0.003)**
IL‐13 TT	0.87 (*P* = 0.53)	**1.92 (*P* = 0.003)**
IL‐4 CR	1.14 (*P* = 0.39)	**3.32 (*P* < 0.0001)**
IL‐4 DM	1.12 (*P* = 0.30)	**2.77 (*P* < 0.0001)**
IL‐4 PHA	**1.45 (*P* = 0.003)**	**1.39 (*P* = 0.009)**
IL‐4 TT	0.93 (*P* = 0.58)	**1.49 (*P* = 0.002)**
IL‐5 CR	1.29 (*P* = 0.25)	**5.87 (*P* < 0.0001)**
IL‐5 DM	1.20 (*P* = 0.28)	**3.40 (*P* < 0.0001)**
IL‐5 PHA	**1.36 (*P* = 0.01)**	**2.20 (*P* < 0.0001)**
IL‐5 TT	1.39 (*P* = 0.07)	**2.26 (*P* < 0.0001)**

Statistically significant associations are denoted in bold.

*The above ratios represent results of general linear models for log‐transformed cytokine response, including both atopy and asthma, controlling for site and cytokine response to media control after 48‐h. Ratio estimates in the asthma column can be interpreted as the ratio of the geometric mean of cytokine response for asthmatics over the geometric mean of cytokine response for non‐asthmatics, while controlling for atopy. Estimates in the atopy column can be interpreted as: the ratio of the geometric mean of cytokine response for atopy over the geometric mean of cytokine response for no atopy, while controlling for current asthma.

There were no group‐related differences in IFN‐γ responses to antigens or polyclonal stimuli (Figure 4 and Appendix Table A1). In the multivariate analysis of innate responses (Appendix Table A1), there were a few associations between innate responses and atopy (e.g., PIC‐induced TNFα and IL‐12p40 and both LPS‐ and PG‐induced IL‐10). Asthma was associated with reductions in RSV‐induced IL‐10 and RV‐induced CXCL8 responses. Contrary to our original hypothesis, asthma was not associated with reductions in virus‐induced IFN‐α or IFN‐γ responses (Appendix Table A1).

## Discussion

Urban and minority populations have increased prevalence and severity of atopy and asthma 2, 24, but there is little information about specific immunologic mechanisms that underlie asthma and allergic disease in the inner‐city population. In this cross‐sectional study, we examined whether atopic or asthmatic status influenced systemic innate and adaptive cytokine responses to antigens, microbial stimuli, viruses, and polyclonal stimuli. We found that allergic sensitization was closely associated with heightened Type 2 immune responses, and these patterns were most pronounced in individuals with antigen‐specific responses to common indoor allergens (cockroach and dust mite). Notably, immune responses of subjects with both asthma and atopy were quite similar to those of subjects with atopy alone. These relationships were true for individuals with atopy in general, or those with allergy to specific indoor allergens such as cockroach and dust mite. These results indicate that increased Type 2 cytokine secretion associated with atopic asthma is mainly related to atopy.

Previous studies conducted in a smaller population including 41 asthmatics and 8 controls with neither asthma nor allergy concluded that PBMC proliferation and cytokine secretion does not differentiate either atopy or asthma from normal individuals 25. However, our results in a larger inner‐city population, suggest that a strong Type 2 bias, particularly in response to allergen stimulation, was detectable in stimulated PBMC cytokine responses of individuals with atopy. These findings are strengthened by the inclusion of a large sample size, which allows for the detection of small differences between groups, as well as inclusion of a large population of women with allergic sensitization but not asthma. Furthermore, our results showing a Th2 bias in atopic subjects in response to antigen are consistent with previous findings in atopic asthmatics 7, 9, 26, 27. The strength of the associations in our study may be related to common exposure and sensitization to potent indoor allergens. Although atopy was associated with changes in PBMC responses, the studies are in relative agreement about the paucity of associations with asthma. These results suggest that compartmentalization of immune abnormalities in atopy and asthma may be distinct.

In our studies, neither atopy nor asthma were associated with impaired virus‐induced interferon responses (IFN‐γ or IFN‐α responses to RSV, RV, or TLR3), which differs from results of several previously published studies 7, 8, 28. The reason for the lack of association could be related to either age or cell source. Impaired Type 1 interferon responses to RVs and RSV, which were not seen in our study population, may be more apparent in early life as compared to the adulthood. In fact, wheezing illnesses in early childhood are associated with reduced blood mononuclear cell IFN‐γ responses in the first year, followed by enhanced IFN‐γ responses thereafter 29. In addition, asthma‐related reductions in antiviral responses asthma may be more pronounced in airway cells exposed to allergic inflammation than in peripheral blood cells, and this has been observed in subjects experimentally inoculated with RV 30. Notably, allergic inflammation can inhibit antiviral responses in vitro. For example, cross‐linked FcϵRI receptors on the surface of dendritic cells inhibit interferon responses to influenza and rhinoviruses 31, 32. This could provide a mechanism for allergen exposure at the respiratory mucosa to inhibit local antiviral responses.

These data should be interpreted within the framework of the strengths and limitations of the study. This study included a fairly comprehensive panel of cytokine responses using assays that were carefully standardized across four study sites 22. Furthermore, our study is unique in that it describes the immune responses in young, urban women with a high prevalence of atopy, and this population has previously been under‐represented in clinical trials. The study is strengthened by its large sample size and by its inclusion of several ethnic minorities, with a predominance of African Americans and Hispanic, living in four geographic sites across the United States. On the other hand, because of these characteristics, the results of this study may not be applicable to all patient populations. We also did not perform formal testing to adjust for multiple comparisons, but instead focused only on consistent findings that exhibited uniform trends across multiple stimuli (e.g., IL‐5 responses to allergens, tetanus and PHA) or multiple responses to a single stimulus (e.g., enhancements of Type 2 responses to cockroach). Finally asthma in this population was predominantly of mild severity, and it is possible that blood cell responses associated with more severe disease could exhibit some unique features.

In conclusion, allergic diseases and asthma cause increased morbidity in urban populations, but the immune mechanisms to explain these observations have been lacking, particularly in adults. Our study of inner‐city women indicates that atopy, potentially driven by sensitization to indoor allergens such as cockroach and dust mite, is associated with a Type‐2‐biased immune profile. However, we detected no asthma‐ or atopy‐related impairment in either IFN‐α or IFN‐γ responses to respiratory viruses or innate immune stimuli. The general lack of specific associations between PBMC responses and asthma may indicate that immunologic abnormalities associated with asthma are largely dependent on environmental exposures and the immunologic milieu of the airways. Additional studies are warranted to determine how airway immune responses are modified by combinations of specific allergies together with environmental exposures that are unique to the urban environment.

## Appendix

**Table:** **Appendix Table A1**. Results from multivariate model relating asthma and atopy to cytokine responses to innate stimulants.*

Cytokine + stimulant	Asthma, *N* = 183/503 (36%)	Atopy, *N* = 342/503 (68%)
IFN‐α PIC	1.05 (*P* = 0.57)	1.07 (*P* = 0.48)
IFN‐α CpG	0.92 (*P* = 0.40)	0.95 (*P* = 0.59)
IFN‐α LPS	0.86 (*P* = 0.08)	0.95 (*P* = 0.54)
IFN‐α PG	1.04 (*P* = 0.22)	0.96 (*P* = 0.19)
IFN‐α RSV	0.89 (*P* = 0.14)	1.07 (*P* = 0.38)
IFN‐α RV	1.23 (*P* = 0.24)	0.86 (*P* = 0.39)
IFN‐γ PIC	1.00 (*P* = 0.99)	1.28 (*P* = 0.14)
IFN‐γ CpG	0.94 (*P* = 0.51)	1.19 (*P* = 0.08)
IFN‐γ LPS	1.04 (*P* = 0.81)	1.08 (*P* = 0.63)
IFN‐γ PG	1.00 (*P* = 0.98)	0.93 (*P* = 0.70)
IFN‐γ RSV	0.90 (*P* = 0.50)	1.08 (*P* = 0.63)
IFN‐γ RV	0.95 (*P* = 0.65)	1.05 (*P* = 0.71)
CXCL8 PIC	0.91 (*P* = 0.28)	1.08 (*P* = 0.38)
CXCL8 CpG	0.87 (*P* = 0.10)	1.12 (*P* = 0.18)
CXCL8 LPS	1.00 (*P* = 0.98)	1.14 (*P* = 0.09)
CXCL8 PG	0.97 (*P* = 0.75)	1.07 (*P* = 0.42)
CXCL8 RSV	0.94 (*P* = 0.49)	1.17 (*P* = 0.09)
CXCL8 RV	**0.80 (*P* = 0.01)**	1.04 (*P* = 0.66)
IL‐10 PIC	0.89 (*P* = 0.23)	1.20 (*P* = 0.06)
IL‐10 CpG	0.96 (*P* = 0.65)	1.06 (*P* = 0.56)
IL‐10 LPS	0.96 (*P* = 0.60)	**1.19 (*P* = 0.05)**
IL‐10 PG	0.93 (*P* = 0.39)	**1.25 (*P* = 0.01)**
IL‐10 RSV	**0.82 (*P* = 0.03)**	1.20 (*P* = 0.05)
IL‐10 RV	0.93 (*P* = 0.49)	1.02 (*P* = 0.84)
IL‐12p40 PIC	1.05 (*P* = 0.72)	**1.34 (*P* = 0.02)**
IL‐12p40 CpG	1.14 (*P* = 0.16)	1.00 (*P* = 0.97)
IL‐12p40 LPS	1.03 (*P* = 0.81)	1.18 (*P* = 0.21)
IL‐12p40 PG	1.17 (*P* = 0.12)	1.11 (*P* = 0.34)
IL‐12p40 RSV	1.08 (*P* = 0.49)	1.15 (*P* = 0.20)
IL‐12p40 RV	1.04 (*P* = 0.69)	1.13 (*P* = 0.23)
TNF‐ α PIC	0.90 (*P* = 0.22)	**1.25 (*P* = 0.01)**
TNF‐ α CpG	0.94 (*P* = 0.43)	1.07 (*P* = 0.35)
TNF‐ α LPS	1.00 (*P* = 0.98)	1.11 (*P* = 0.11)
TNF‐ α PG	1.03 (*P* = 0.71)	1.11 (*P* = 0.26)
TNF‐ α RSV	0.85 (*P* = 0.12)	1.13 (*P* = 0.24)
TNF‐ α RV	0.89 (*P* = 0.18)	0.99 (*P* = 0.91)

Statistically significant associations are denoted in bold.

* The above ratios represent results of general linear models for log‐transformed cytokine response, including both atopy and asthma, controlling for site. Ratio estimates in the asthma column can be interpreted as the ratio of the geometric mean of cytokine response for asthmatics over the geometric mean of cytokine response for non‐asthmatics, while controlling for atopy. Estimates in the atopy column can be interpreted as: the ratio of the geometric mean of cytokine response for atopy over the geometric mean of cytokine response for no atopy, while controlling for current asthma.

## References

[1] Gergen, P. J , and K. B. Weiss . 1990 Changing patterns of asthma hospitalization among children: 1979 to 1987. JAMA 264:1688–1692. 2398608

[2] Togias, A. , M. J Fenton , P. J. Gergen , D. Rotrosen , and A. S. Fauci . 2010 Asthma in the inner city: the perspective of the National Institute of Allergy and Infectious Diseases. J. Allergy Clin. Immunol. 125:540–544. 20226290 10.1016/j.jaci.2010.01.040

[3] Evans R., 3rd , D. I. Mullally , R. W. Wilson , P. J. Gergen , H. M. Rosenberg , J. S. Grauman , F. M. Chevarley , and M. Feinleib . 1987 National trends in the morbidity and mortality of asthma in the US. Prevalence, hospitalization and death from asthma over two decades: 1965–1984. Chest 91:65S–74S. 3581966

[4] Prescott, S. L. , P. Noakes , B. W. Chow , L. Breckler , C. A. Thornton , E. M. Hollams , M. Ali , A. H. van den Biggelaar , and M. K. Tulic . 2008 Presymptomatic differences in Toll‐like receptor function in infants who have allergy. J. Allergy Clin. Immunol. 122:391–399, 9 e1–e5. 18571707 10.1016/j.jaci.2008.04.042

[5] Sumino, K. , J. Tucker , M. Shahab , K. F. Jaffee , C. M. Visness , J. E. Gern , G. R. Bloomberg , and M. J. Holtzman . 2012 Antiviral IFN‐gamma responses of monocytes at birth predict respiratory tract illness in the first year of life. J. Allergy Clin. Immunol. 129:1267–1273, e1. 22460071 10.1016/j.jaci.2012.02.033 PMC3340511

[6] Hollams, E. M. , M. Deverell , M. Serralha , D. Suriyaarachchi , F. Parsons , G. Zhang , N. de Klerk , B. J. Holt , C. Ladyman , A. Sadowska , et al. 2009 Elucidation of asthma phenotypes in atopic teenagers through parallel immunophenotypic and clinical profiling. J. Allergy Clin. Immunol. 124:463–470, 70 e1–16. 19733295 10.1016/j.jaci.2009.06.019

[7] Smart, J. M. , E. Horak , A. S. Kemp , C. F. Robertson , and M. L. Tang . 2002 Polyclonal and allergen‐induced cytokine responses in adults with asthma: resolution of asthma is associated with normalization of IFN‐gamma responses. J. Allergy Clin. Immunol. 110:450–456. 12209093 10.1067/mai.2002.127283

[8] Brooks, G. D. , K. A. Buchta , C. A. Swenson , J. E. Gern , and W. W. Busse . 2003 Rhinovirus‐induced interferon‐gamma and airway responsiveness in asthma. Am. J. Respir. Crit. Care Med. 168:1091–1094. 12928311 10.1164/rccm.200306-737OC

[9] Zoratti, E. , S. Havstad , S. G. Wegienka , C. Nicholas , K. R. Bobbitt , K. J. Woodcroft , D. R. Ownby , and C. C. Johnson . 2014 Differentiating asthma phenotypes in young adults through polyclonal cytokine profiles. Ann. Allergy Asthma Immunol. 113:25–30. 24801891 10.1016/j.anai.2014.04.013 PMC4065816

[10] Nurse, B. , A. S. Puterman , M. Haus , D. Berman , E. G. Weinberg , and P. C. Potter . 2000 PBMCs from both atopic asthmatic and nonatopic children show a TH2 cytokine response to house dust mite allergen. J. Allergy Clin. Immunol. 106:84–91. 10887310 10.1067/mai.2000.107397

[11] Barnes, P. J. 2008 The cytokine network in asthma and chronic obstructive pulmonary disease. J. Clin. Invest. 118:3546–3556. 18982161 10.1172/JCI36130 PMC2575722

[12] Lemanske R. F., Jr. , and W. W. Busse . 2010 Asthma: clinical expression and molecular mechanisms. J. Allergy Clin. Immunol. 125:S95–102. 20176271 10.1016/j.jaci.2009.10.047 PMC2853245

[13] Holt, P. G. , D. H. Strickland , A. Bosco , and F. L. Jahnsen . 2009 Pathogenic mechanisms of allergic inflammation: atopic asthma as a paradigm. 104:51–113. 10.1016/S0065-2776(08)04003-0 20457116

[14] Holt, P. G. , and D. H. Strickland . 2010 Interactions between innate and adaptive immunity in asthma pathogenesis: new perspectives from studies on acute exacerbations. J. Allergy Clin. Immunol. 125:963–972; quiz 73–74. 20394979 10.1016/j.jaci.2010.02.011

[15] Jackson, D. J. , M. D. Evans , R. E. Gangnon , C. J. Tisler , T. E. Pappas , W. M. Lee , J. E. Gern , and R. F. Lemanske, Jr. 2012 Evidence for a causal relationship between allergic sensitization and rhinovirus wheezing in early life. Am. J. Respir. Crit. Care Med. 185:281–285. 21960534 10.1164/rccm.201104-0660OC PMC3297109

[16] Kusel, M. M. , N. H. de Klerk , T. Kebadze , V. Vohma , P. G. Holt , S. L. Johnston , and P. D. Sly . 2007 Early‐life respiratory viral infections, atopic sensitization, and risk of subsequent development of persistent asthma. J. Allergy Clin. Immunol. 119:1105–1110. 17353039 10.1016/j.jaci.2006.12.669 PMC7125611

[17] Holt, P. G. , and P. D. Sly . 2011 Interaction between adaptive and innate immune pathways in the pathogenesis of atopic asthma: operation of a lung/bone marrow axis. Chest 139:1165–1171. 21540215 10.1378/chest.10-2397

[18] Baraldo, S. , M. Contoli , E. Bazzan , G. Turato , A. Padovani , B. Marku , F. Calabrese , G. Caramori , A. Ballarin , D. Snijders , et al. 2012 Deficient antiviral immune responses in childhood: distinct roles of atopy and asthma. J. Allergy Clin. Immunol. 130:1307–1314. 22981791 10.1016/j.jaci.2012.08.005

[19] Contoli, M. , S. D. Message , V. Laza‐Stanca , M. R. Edwards , P. A. Wark , N. W. Bartlett , T. Kebadze , P. Mallia , L. A. Stanciu , H. L. Parker , et al. 2006 Role of deficient type III interferon‐lambda production in asthma exacerbations. Nat. Med. 12:1023–1026. 16906156 10.1038/nm1462

[20] Wark, P. A. , S. L. Johnston , F. Bucchieri , R. Powell , S. Puddicombe , V. Laza‐Stanca , S. T. Holgate , and D. E. Davies . 2005 Asthmatic bronchial epithelial cells have a deficient innate immune response to infection with rhinovirus. J. Exp. Med. 201:937–947. 15781584 10.1084/jem.20041901 PMC2213100

[21] Gern, J. E. , C. M. Visness , P. J. Gergen , R. A. Wood , G. R. Bloomberg , G. T. O'Connor , M. Kattan , H. A. Sampson , F. R. Witter , M. T. Sandel , et al. 2009 The Urban Environment and Childhood Asthma (URECA) birth cohort study: design, methods, and study population. BMC Pulm. Med. 9:17. 19426496 10.1186/1471-2466-9-17 PMC2689166

[22] Shreffler, W. G. , C. M. Visness , M. Burger , W. W. Cruikshank , H. M. Lederman , M. de la Morena , K. Grindle , A. Calatroni , H. A. Sampson , and J. E. Gern . 2006 Standardization and performance evaluation of mononuclear cell cytokine secretion assays in a multicenter study. BMC Immunol. 7:29. 17156490 10.1186/1471-2172-7-29 PMC1762025

[23] Gold, D. R. , G. R. Bloomberg , W. W. Cruikshank , C. M. Visness , J. Schwarz , M. Kattan , G. T. O'Connor , R. A. Wood , M. S. Burger , R. J. Wright , et al. 2009 Parental characteristics, somatic fetal growth, and season of birth influence innate and adaptive cord blood cytokine responses. J. Allergy Clin. Immunol. 124:1078–1087. 19895995 10.1016/j.jaci.2009.08.021 PMC2796683

[24] Matsui, E. C. , N. N. Hansel , M. C. McCormack , R. Rusher , P. N. Breysse , and G. B. Diette . 2008 Asthma in the inner city and the indoor environment. Immunol. Allergy Clin. North Am. 28:665–686. 18572113 10.1016/j.iac.2008.03.004 PMC5516633

[25] Simms, E. , M. Kjarsgaard , S. Denis , F. E. Hargreave , P. Nair , and M. Larche . 2013 Cytokine responses of peripheral blood mononuclear cells to allergen do not identify asthma or asthma phenotypes. Clin. Exp. Allergy 43:1226–1235. 24152155 10.1111/cea.12194

[26] Kenyon, N. , E. Kelly , and N. Jarjour . 2000 Enhanced cytokine generation by peripheral blood mononuclear cells in allergic and asthma subjects. Ann. Allergy Asthma Immunol. 85:115–120. 10982218 10.1016/S1081-1206(10)62450-7

[27] Nieves, A. , A. Magnan , S. Boniface , H. Proudhon , A. Lanteaume , S. Romanet , D. Vervloet , and P. Godard . 2005 Phenotypes of asthma revisited upon the presence of atopy. Respir. Med. 99:347–354. 15733511 10.1016/j.rmed.2004.08.004

[28] Busse, W. W. , R. F. Lemanske, Jr. , and J. E. Gern . 2010 Role of viral respiratory infections in asthma and asthma exacerbations. Lancet 376:826–834. 20816549 10.1016/S0140-6736(10)61380-3 PMC2972660

[29] Uekert, S. J. , G. Akan , M. D. Evans , Z. Li , K. Roberg , C. Tisler , D. Dasilva , E. Anderson , R. Gangnon , D. B. Allen , et al. 2006 Sex‐related differences in immune development and the expression of atopy in early childhood. J. Allergy Clin. Immunol. 118:1375–1381. 17157669 10.1016/j.jaci.2006.09.008

[30] Sykes, A. , M. R. Edwards , J. Macintyre , A. del Rosario , E. Bakhsoliani , M. B. Trujillo‐Torralbo , O. M. Kon , P. Mallia , M. McHale , and S. L. Johnston . 2012 Rhinovirus 16‐induced IFN‐alpha and IFN‐beta are deficient in bronchoalveolar lavage cells in asthmatic patients. J. Allergy Clin. Immunol. 129:1506–1514, e6. 22657407 10.1016/j.jaci.2012.03.044

[31] Gill, M. A. , G. Bajwa , T. A. George , C. C. Dong , I. I. Dougherty , N. Jiang , V. N. Gan , and R. S. Gruchalla . 2010 Counterregulation between the FcepsilonRI pathway and antiviral responses in human plasmacytoid dendritic cells. J. Immunol. 184:5999–6006. 20410486 10.4049/jimmunol.0901194 PMC4820019

[32] Durrani, S. R. , D. J. Montville , A. S. Pratt , S. Sahu , M. K. DeVries , V. Rajamanickam , R. E. Gangnon , M. A. Gill , J. E. Gern , R. F. Lemanske, Jr. , et al. 2012 Innate immune responses to rhinovirus are reduced by the high‐affinity IgE receptor in allergic asthmatic children. J. Allergy Clin. Immunol. 130:489–495. 22766097 10.1016/j.jaci.2012.05.023 PMC3437329

